# Supplementary material for: [18F]FDG PET/CT in Large Vessel Vasculitis: The Impact of Expertise and Confounders on Image Analysis
Source: Diagnostics (Basel). 2022 Nov 7;12(11):2717. doi: 10.3390/diagnostics12112717 (PMC9689655; doi:10.3390/diagnostics12112717)
Supplement: Supplementary file 1 [file diagnostics-12-02717-s001.zip › diagnostics-1975488-supplementary.pdf]

Article

[18F]FDG PET/CT in Large Vessel Vasculitis: The Impact of Expertise and Confounders on Image Analysis

Lidija Antunovic [1], Alessia Artesani [1,2], Michael Coniglio [2], Wim J. G. Oyen [2,3], Michele Ciccarelli [1], Carlo Selmi [1,2], Arturo Chiti [1,2], Martina Sollini [1,2,*]

1 IRCCS Humanitas Research Hospital, Via Manzoni 56, Rozzano, 20089 Milan, Italy
2 Department of Biomedical Sciences, Humanitas University, Via Rita Levi Montalcini 4, Pieve Emanuele, 20090 Milan, Italy
3 Department of Radiology and Nuclear Medicine, Rijnstate Hospital, 6815 AD Arnhem, The Netherlands
* Correspondence: martina.sollini@hunimed.eu

Table S1: Interrater agreement between reader 1 and reader 2.

Interrater agreement of the diagnostic performances of the student compared to the diagnostic performances of the experienced nuclear medicine physician, detailing the values of sensitivity, specificity, accuracy, PPN, NPV, and Cohen's Kappa overall and in each subset of patients.

Population	Age		BMI		Steroids		Glycaemia		Interval Injection-Acquisition			PET Scanner	
	≤65	>65	Normal	Over	Yes	No	≤126	>126	<60	60-65	>65	P1	P2
Sensitivity	66.3	83.9	55.8	75.6	55.3	67.3	64.5	65.7	69.2	75.8	56.5	63.0	31.3
Specificity	73.5	73.7	73.3	66.7	80.0	73.5	73.5	71.8	84.6	80.0	73.2	66.2	92.4
Accuracy	71.3	76.6	68.0	69.5	73.2	71.3	71.3	70.0	79.5	78.7	68.4	65.2	82.1
PPV	51.4	56.5	47.5	51.5	51.2	57.4	43.5	48.9	69.2	62.5	46.4	43.6	45.5
NPV	83.7	91.8	79.3	85.3	82.5	80.9	86.7	83.6	84.6	88.2	80.4	81.1	86.9
**Cohen's Kappa coefficient**													
Kappa	0.37	0.50	0.28	0.38	0.34	0.39	0.33	0.34	0.54	0.53	0.28	0.26	0.27

BMI: body mass index; NPV: negative predictive value; P1: PET 1; P2: PET 2; PET: positron emission tomography; PPV: positive predictive value

Table S2. Semi-quantitative PET evaluation.

Mean values of each anatomical region of interest for both patients with and without vasculitis on semi-quantitative evaluation.

	Patients with vasculitis (mean±SD)	Patients without vasculitis (mean±SD)	p-value
Left carotid	2.12±0.54	2.08±0.57	0.4725
Right carotid	2.19±0.56	2.14±0.56	0.3376
Left subclavian	1.80±0.59	1.71±0.57	0.1113
Right subclavian	1.87±0.62	1.77±0.56	0.1238
Left axillary	1.71±0.79	1.50±0.60	0.0395
Right axillary	1.77±0.8	1.54±0.60	0.0321
Ascending aorta	2.70±0.66	2.55±0.59	0.0633
Aortic arch	2.66±0.51	2.57±0.57	0.1059
Descending aorta	2.53±0.46	2.55±0.55	0.9849
Abdominal aorta	2.78±0.75	2.67±0.64	0.3562
Left iliac	2.43±0.65	2.30±0.58	0.1651

Right iliac	2.41±0.66	2.35±0.63	0.6906
Left femoral	1.96±0.51	1.93±0.56	0.5421
Right femoral	2.12±0.54	2.08±0.57	0.4725
Liver	2.19±0.56	2.14±0.56	0.3376
IVC	1.80±0.59	1.71±0.57	0.1113

IVC: inferior vena cava; SD: standard deviation

Table S3. Comparison between qualitative and semi-quantitative PET evaluation.

Diagnostic performances of the experienced nuclear medicine physician (reader 1) and the student (reader 2). detailing the values of sensitivity. specificity. accuracy. PPN. NPV. with and without liver and IVC cut-off.

	**Reader 1**			**Reader 2**		
	**Qualitative**	**Liver cut-off**	**IVC cut-off**	**Qualitative**	**Liver cut-off**	**IVC cut-off**
Sensitivity	54.3	59.3	92.6	59.3	61.7	92.6
Specificity	80.3	72.7	17.2	70.2	63.6	14.6
Accuracy	72.8	68.8	39.1	67.0	63.1	37.3
PPV	53.0	47.1	31.4	44.9	41.0	30.7
NPV	81.1	81.4	85.0	80.8	80.3	82.9

IVC: inferior vena cava; NPV: negative predictive value; PPV: positive predictive value

Table S4. Timing suggested by EARL guidelines.

Number of true positive. true negative. false positive and false negative exams for both readers. considering timing suggested by EARL guidelines.

	<55 min		55-75 min		>75 min	
	**Reader 1**	**Reader 2**	**Reader 1**	**Reader 2**	**Reader 1**	**Reader 2**
True positive	5	5	34	39	5	4
True negative	21	21	127	113	11	5
False positive	5	5	30	44	4	10
False negative	5	5	30	25	2	3
Total	36		221		22	
